# Freeze-all cycle in reproductive medicine: current perspectives

**DOI:** 10.5935/1518-0557.20170012

**Published:** 2017

**Authors:** Matheus Roque, Marcello Valle, Alessandra Kostolias, Marcos Sampaio, Selmo Geber

**Affiliations:** 1 ORIGEN – Center for Reproductive Medicine, Rio de Janeiro, Brazil; 2 UFMG, Federal University of Minas Gerais, Belo Horizonte, Brazil; 3 ORIGEN – Center for Reproductive Medicine, Belo Horizonte, Brazil

**Keywords:** freeze-all, elective frozen-thawed embryo transfer, delayed frozen-thawed embryo transfer, cryopreservation, IVF

## Abstract

The freeze-all strategy has emerged as an alternative to fresh embryo transfer (ET) during *in vitro* fertilization (IVF) cycles. Although fresh ET is the norm during assisted reproductive therapies (ART), there are many concerns about the possible adverse effects of controlled ovarian stimulation (COS) over the endometrium. The supra-physiologic hormonal levels that occur during a conventional COS are associated with modifications in the peri-implantation endometrium, which may be related to a decrease in pregnancy rates and poorer obstetric and perinatal outcomes when comparing fresh to frozen-thawed embryo transfers. The main objective of this study was to assess the available literature regarding the freeze-all strategy in IVF cycles, in regards to effectiveness and safety. Although there are many potential advantages in performing a freeze-all cycle over a fresh ET, it seems that the freeze-all strategy is not designed for all IVF patients. There is a need to develop a non-invasive clinical tool to evaluate the endometrial receptivity during a fresh cycle, which enables the selection of patients that would benefit from this strategy. Today, it is reasonable to perform elective cryopreservation of all oocytes/embryos in cases with a risk of OHSS development, and in patients with supra-physiologic hormonal levels during the follicular phase of COS. It is not clear if all normal responders and poor responders may benefit from this strategy.

## INTRODUCTION

Although fresh ET is the norm during assisted reproductive therapies (ART), in the past few years, the freeze-all strategy has emerged as an alternative to fresh embryo transfer (ET) during in vitro fertilization (IVF) cycles (Roque, 2015a). Controlled ovarian stimulation (COS) is necessary for the development and maturation of many follicles and oocytes, thus increasing the likelihood of positive outcomes and cumulative pregnancy rates during ART (Siristatidis *et al*., 2013). However, there are many concerns about the possible adverse effects of controlled ovarian stimulation (COS) over the endometrium. The supra-physiological hormonal levels that occur during a conventional COS are associated with modifications in the peri-implantation endometrium, that may be related to decreases in pregnancy rates (Shapiro *et al*., 2011a; Roque *et al*., 2013), and poorer obstetric and perinatal outcomes (Maheshwari *et al*., 2012; Pandey *et al*., 2012; Pinborg *et al*., 2013), when comparing fresh to frozen-thawed embryo transfers.

In the freeze-all strategy, the entire cohort of embryos is cryopreserved (not just the "second best"), and the best embryos are transferred in a later cycle into a more physiologic endometrium (Roque, 2015a). IVF success depends not only on embryo quality, but also on endometrial receptivity and on the embryo-endometrium interaction, these modifications in uterine environment, found during COS, may jeopardize the IVF outcomes after fresh embryo transfer, when compared to FET. By performing delayed frozen-thawed ET (FET), the deleterious effects of COS over the endometrium would be avoided, and better outcomes could be expected (Shapiro *et al*., 2011a; Roque *et al*., 2013; Roque, 2015a).

The main objective of this study was to assess the available literature regarding the effectiveness and safety of the freeze-all strategy in IVF cycles.

### The rationale of freeze-all cycles

There is scientific evidence showing that COS may be related to endometrial advancement, which can be seen during histological evaluation in a fresh IVF cycle (Ubaldi *et al*., 1997; Kolibianakis *et al*., 2002). In 1997 Ubaldi *et al*. performed endometrial biopsies during a fresh cycle and evaluated the histological dating. They reported that when the endometrial advancement was over 3 days, no pregnancy was achieved. All these patients had progesterone (*p*) levels ≥ 1.1ng/mL on the trigger day. The mean number of retrieved oocytes in this group of patients was 15.8. In the group of patients with lower *p* levels, the endometrial advancement was of 3 days or less, suggesting no interference of ovarian stimulation over the endometrium in this group of patients (Ubaldi *et al*., 1997). These findings were later corroborated by the study carried out by Kolibianakis *et al*. (2002).

The technique used to evaluate the endometrium has evolved, and in 2005 Horcajadas *et al*. published a study evaluating the endometrium gene expression profile. They performed endometrial biopsies in the same oocyte donors during a fresh cycle on the 7^th^ day after LH surge, and compared it to endometrial samples on the 7^th^ day after hCG trigger in a stimulated cycle. They found that there were over 200 genes related to implantation that were over or under expressed during COS, when compared to a natural cycle. These changes may be associated with the supra-physiologic hormonal levels observed during COS. Labarta *et al*. (2011) found differences in endometrial gene expression between patients with elevated *p* (≥ 1.1ng/mL) on the day of final oocyte maturation, when compared with patients with normal *p* levels. Van Vaerenbergh *et al*. (2009) showed a correlation between endometrial dating by Noyes' criteria and endometrial gene expression. They also found that patients that had endometrial advancement of more than 3 days did not get pregnant, and they correlated these histological findings with the gene expression profile.

The aforementioned studies suggested that hyperstimulation might be detrimental to implantation, by altering genes that are crucial for the endometrium-embryo interaction. However, all altered findings (histological and gene expression profile) were found in patients with normal to high ovarian response (Ubaldi *et al*., 1997; Kolibianakis *et al*., 2002; Horcajadas *et al*., 2005; Labarta *et al*., 2011). For example, in the protocol implemented by Horcajadas *et al*. (2005), COS resulted in the retrieval of 13-18 oocytes and an average E_2_ level of 2200 pg./mL. Thus, until now, we cannot extrapolate these findings to patients with all subtypes of ovarian response.

These modifications in the endometrium, occurring because of COS, may have consequences not only on implantation rates during IVF treatments, but also be associated with obstetric and perinatal complications. Epidemiologic studies suggest that the maternal peri-implantation environment plays a critical role in perinatal outcomes (Mainigi *et al*., 2014). It was shown in animal models that the altered hormonal milieu related to gonadotropin administration plays a critical role and has a direct effect over fetal growth, trophoblast differentiation, and gene expression. The exact cellular and molecular mechanisms are not well established. However, it seems that altered trophoblast expansion and invasion is, at least in part, responsible for the consequences (Mainigi *et al*., 2014).

### Effectiveness

**Figure 1:**
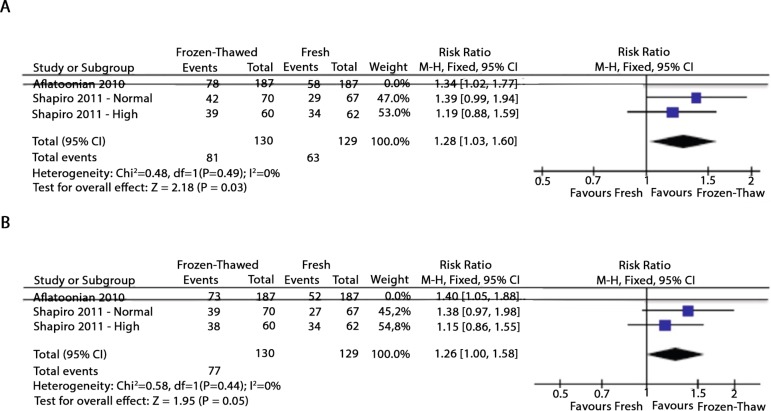
Forest plot of elective frozen-thawed embryo transfers versus fresh embryo transfers: A - Clinical pregnancy rate; and B – Ongoing pregnancy rate.

A recent meta-analysis (Roque *et al*., 2013) showed an increase of 32% in the ongoing pregnancy rate when the freeze-all strategy was performed, when compared to fresh ET^4^. However, there were only three studies included in this meta-analysis (Aflatoonian *et al*., 2010; Shapiro *et al*., 2011a, Shapiro *et al*., 2011b), and one of them (Aflatoonian *et al*., 2010) was recalled due to methodological problems. We performed another analysis excluding the aforementioned study, as shown in Figure 1. The analysis of the available data also showed that eFET resulted in a statistically significant increase in the clinical pregnancy rate when compared to the rate found with fresh embryo transfer (RR = 1.28, 95% confidence interval [CI]: 1.03–1.60; I^2^ = 0%) (Figure 1A). Nonetheless, when we evaluated ongoing pregnancy rates (OPR), the eFET group showed a higher OPR compared to the fresh embryo transfer group, but this difference did not reach statistical significance (RR = 1.26, 95% CI: 1.00–1.58; I^2^ = 0%) (Figure 1B). This new analysis included 259 in vitro fertilization (IVF) cycles in normal and high responders following blastocyst embryo transfers, considering two studies from the same reproductive center (Fig 1). This data has been already published in the comments of the previous published study (Roque *et al*., 2013) on the Fertility and Sterility website.

Ferrareti *et al*. (1999) published a study comparing elective cryopreservation of all embryos to fresh embryo transfer in patients with risk of OHSS development. They did not find any benefit in delaying ET. However, this was a study from 17 years ago, and these findings may be associated with the cryopreservation techniques used in 1999 and not with the strategy per se. A randomized clinical trial (RCT) (Chen *et al*., 2016) was recently published, evaluating 1508 infertile women with polycystic ovary syndrome, who were submitted to a first IVF cycle, comparing fresh embryo transfer to elective FET. The patients in the freeze-all group had a higher frequency of live births after the first transfer, when compared to fresh embryo transfers (49.3% *vs*. 42.0%, *p* = 0.004). These results were mainly due to a lower frequency of pregnancy loss in the freeze-all group (RR = 0.67; 95% CI 0.54-0.83; *p* < 0.001). Thus, until now, there are only three recent RCT concerning the freeze-all strategy.

All previous studies evaluating this strategy were performed in patients with normal/high ovarian response (Shapiro *et al*., 2011a; Shapiro *et al*., 2011b; Roque *et al*., 2015c; Braga *et al*., 2016; Chen *et al*., 2016). Therefore, it is not possible to extrapolate the results to all kinds of ovarian response, such as poor ovarian responders (POR). Until now, there are no studies evaluating this strategy in POR. Moreover, these studies showed that although this strategy would lead to improvements in IVF outcomes of at least 30% in CPR and OPR when compared to fresh embryo transfers (Shapiro *et al*., 2011a; Shapiro *et al*., 2011b; Roque *et al*., 2015c; Braga *et al*., 2016; Chen *et al*., 2016), there are also many patients that get pregnant even after fresh embryo transfers. To date, there is no effective non-invasive clinical tool to evaluate ER. This tool would help select those patients without alterations in ER that should be maintained in the fresh embryo transfer, and select patients that really benefit from the freeze-all strategy.

More randomized clinical trials (RCT) evaluating this strategy are necessary, and not only in normal and high responders. There are some registered RCT aiming to evaluate this strategy (NCT00823121, NCT02148393, NCT02471573, NTR3187, ACTRN 12612000422820, HTA 13/115/82), and we will probably have more robust evidence when these studies are concluded.

### Safety

When elective FET was implemented, the main idea was to improve IVF outcomes. However, it is important to evaluate not only the effectiveness of ART, but also its safety. One of the major complications observed during COS in IVF cycles is ovarian hyperstimulation syndrome (OHSS). It is iatrogenic, potentially lethal and occurs in approximately 1%-14% of ART cycles (Nastri *et al*., 2010). Nowadays, it is fundamental to prevent the development of OHSS. When the final oocyte maturation with GnRH agonist is performed in patients with an antagonist protocol and all oocytes/embryos are cryopreserved, the onset of early and late OHSS is virtually eliminated (Devroey *et al*., 2011; Kol & Humaidan, 2013).

The uterine environment during fresh embryo transfers differs from that during FET. In a stimulated cycle, there may be an increase in uterine contractility and embryo-endometrium asynchrony. This would lead to an increase in the risk of ectopic pregnancy (EP) when comparing fresh transfer to FET (Huang *et al*., 2014; Londra *et al*., 2015). Moreover, there is lower risk of low birth weight (Pinborg *et al*., 2010; Li *et al*., 2014; Ishihara *et al*., 2014), pre-term birth (Pelkonen *et al*., 2010; Pinborg *et al*., 2013; Wennerholm *et al*., 2013; Ishihara *et al*., 2014; Schwarze *et al*., 2015), small for gestational age (Ishihara *et al*., 2014; Li *et al*., 2014; Pinborg *et al*., 2014) after FET when comparing to fresh ET. However, the FET cycles are associated with a higher incidence of large for gestational age (Wennerholm *et al*., 2013; Pinborg *et al*., 2014), and higher risk of placenta accreta (Ishihara *et al*., 2014; Kaser *et al*., 2015). It is still controversial if the risk of hypertensive disorders is increased or not among FET patients when compared to their fresh cycles counterparts.

### Cost-effectiveness

There is a lack of studies evaluating the cost-effectiveness of the freeze-all policy. To our knowledge, there is only one published study evaluating this (Roque *et al*., 2015b). The authors concluded that this strategy was cost-effective when compared to fresh embryo transfers. More studies are necessary to evaluate the incremented costs when performing elective cryopreservation of all embryos.

## CONCLUSION

Although there are many potential advantages in performing a freeze-all cycle over a fresh ET, it seems that the freeze-all strategy is not suited for all IVF patients. There are many patients that get pregnant and don't have any obstetrical/perinatal complication even after fresh ET. Moreover, there is a need for studies comparing the costs and cumulative pregnancy rates between the two strategies. There is a need to develop a non-invasive clinical tool to evaluate endometrial receptivity during a fresh cycle, which may enable the selection of patients that would benefit from this strategy. To date, it is reasonable to perform elective cryopreservation of all oocytes/embryos in cases with a risk of OHSS development and in patients with supra-physiologic hormonal levels during the follicular phase of COS. It is not clear if all normal responders and poor responders may benefit from this strategy. All other cases should be discussed with the patients, evaluating the pros and cons, including potential costs, delays in treatment, and potential risks associated with this strategy.
